# Profiling complement proteins in pediatric tuberculosis children with or without serological evidence of prior SARS-CoV-2 exposure

**DOI:** 10.3389/fimmu.2026.1761877

**Published:** 2026-08-07

**Authors:** Nathella Pavan Kumar, Guruprashath Mohan, Sarath Balaji, Agalya Vanamudhu, Prabhakar R, Balaji Ramraj, Poorna Ganga Devi, Shaik Fayaz Ahamed, Karthik M, Gopika S, Sravan Kumar Adavath, Hema Giranab C, Poovazhagi Varadarajan, Subash Babu, Aishwarya Venkataraman

**Affiliations:** 1 ICMR - National Institute for Research in Tuberculosis, Chennai, India; 2 Institute of Child Health and Hospital for Children, Chennai, India; 3 Government Rajaji Hospital, Madurai, India; 4 National Institutes of Health - International Center for Excellence in Research, Chennai, India; 5 Laboratory of Parasitic Diseases, National Institute of Allergy and Infectious Diseases, National Institutes of Health, Bethesda, MA, United States

**Keywords:** Pediatric TB, SARS-CoV-2, COVID-19, complement proteins, multiplex ELISA

## Abstract

**Introduction:**

Coinfection with *Mycobacterium tuberculosis* (TB) and SARS-CoV-2 poses unique challenges in pediatric population due to overlapping immune pathways and potential immune dysregulation. The complement system, a key arm of innate immunity, plays a dual role in pathogen clearance and inflammation.

**Methods:**

This study aimed to investigate complement activation in TB in children with or without serological evidence of prior SARS-CoV-2 exposure. Children aged 2–17 years with pulmonary or extrapulmonary TB were recruited from two tertiary hospitals in South India. Participants were grouped based on SARS-CoV-2 IgG serostatus: CoV2⁺ (n = 30) and CoV2⁻ (n = 30). Blood samples were collected at baseline, and at months 3, and 6 for complement profiling. Levels of classical, lectin, and alternative pathway proteins, as well as regulatory factors, were quantified using multiplex bead-based assays. Canonical discriminant analysis (CDA) and Spearman correlation were used to analyse temporal and relational patterns in complement activation.

**Results:**

CoV2^+^ children had significantly elevated levels of classical pathway proteins (C1q, C3, C4, C5a) and regulatory factors (Factor B, H, and I) at baseline, month 3 and month 6 in comparison to CoV2^-^ children. However C2 and C5 diminished in CoV2^+^ children in comparison to CoV2-CDA revealed distinct, time-resolved complement activity in CoV2⁻ children, whereas CoV2⁺ profiles showed overlap, suggesting prolonged immune alteration. Correlation analysis identified significant associations between SARS-CoV-2 IgG and complement proteins, with differing patterns in CoV2^+^ and CoV2^-^ groups over time.

**Discussion:**

SARS-CoV-2 seropositivity may contribute to changes in the complement activation trajectory in TB-diseased children, potentially contributing to persistent inflammation. Complement profiling may inform therapeutic strategies and serve as a biomarker of immune recovery in TB– SARS-CoV-2 co-infection.

## Introduction

Pediatric tuberculosis, although frequently neglected, constitutes a crucial element in the transmission dynamics and public health management of tuberculosis (TB). Children are especially susceptible to severe form of tuberculosis and serve as a reservoir for future disease transmission, hence rendering pediatric tuberculosis a priority in worldwide eradication initiatives (1). Since its emergence in late 2019, SARS-CoV-2 has complicated the landscape of infectious diseases. As the causative agent of COVID-19, it has had a profound global impact on TB management by increasing morbidity and mortality while also disrupting TB care services (2, 3). SARS-CoV-2 seropositivity which reflects prior exposure is defined by the presence of anti-SARS-CoV-2 antibodies and absence of clinical symptoms, it may indicate underlying immune activation. The pediatric complement response to SARS-CoV-2 infection is distinct from adults and includes complex cytokine signaling and innate immune activation (4). Children tend to have milder disease manifestations, possibly due to differential viral receptor expression, more effective innate immunity (5).

TB and SARS-CoV-2 exhibit a primary tropism for the lungs, which raises substantial concerns regarding the implications of coinfection. Increasing evidence suggests that co-infection with TB and SARS-CoV-2 is not only additive but potentially detrimental in a synergistic manner (6, 7). Clinical data from TB-endemic regions reveal that SARS-CoV-2 infection might precede, follow, or co-occur with TB, which might likely affect the disease progression and treatment outcome. Despite this, the immunopathogenesis of TB–COVID-19 coinfection is not well-defined (6).

Understanding the immunopathogenesis of TB and SARS-CoV-2 coinfection reveals crucial insights into host-pathogen interactions and immune dysregulation. Mapping shared and distinct immune pathways can guide therapeutic strategies (8). The complement system, a key component of innate immunity, plays a dual role—facilitating pathogen clearance and contributing to inflammation. Both viral (e.g., SARS-CoV-2 spike and nucleocapsid proteins) and bacterial pathogens (e.g., *Mycobacterium tuberculosis*) can activate the complement cascade, which supports immune defense but, if uncontrolled, can lead to tissue damage (9).

Our previous studies demonstrated that complement activity is significant in pediatric SARS-CoV-2 cases and predictive of poor TB outcomes (10). This supports our central hypothesis: Modulating the complement system may offer a novel therapeutic approach to mitigate the severity and long-term effects of TB–SARS-CoV-2 coinfection in children—potentially improving lung function, slowing TB progression, and reducing the risk of severe COVID-19.

## Materials and methods

### Ethics statement

Informed consent was received from the parents or guardians of all participating children, with assent from the children where in appropriate. The Internal Ethics Committee (IEC) of the ICMR-National Institute for Research in Tuberculosis, along with the participating institutions (Institute of Child Health, Chennai and Madurai Medical College, Madurai), approved the study.

### Study population and procedures

This research built upon the TB-COVID study and involved children between the ages of 2 to 17 years who were diagnosed with either pulmonary tuberculosis (PTB) or extrapulmonary tuberculosis (EPTB); this included both confirmed TB as well those diagnosed clinically, from the Institute of Child Health in Chennai and Government Rajaji Hospital in Madurai. Excluded from the study were children with central nervous system TB or disseminated TB or those who required anti-tuberculous treatment for longer than six months. All participants were subjected to SARS-CoV-2 RT-PCR testing upon enrolment, and blood samples were collected for SARS-CoV-2 IgG assay. Depending on their anti-SARS-CoV-2 IgG results, the children were divided into two categories: TB with a positive SARS-CoV-2 antibody status [CoV2^+^] (n = 30) and TB with a negative SARS-CoV-2 antibody status [CoV2^-^] (n = 30). In all children, blood samples were collected at baseline (prior to anti-tuberculosis treatment) and at 3 months (during treatment) and 6 months (after treatment completion). The collected blood samples were transported to the ICMR-National Institute for Research in Tuberculosis (NIRT) in Chennai and Madurai and processed within 4 h of collection. The samples were centrifuged, and the plasma was stored in freezers at −80 °C until it was analyzed. The demographic and epidemiological data (**Table 1**) (11) have been previously reported.

**Table 1:** Demographic data of study population.

S.No	Characteristics of study population	TB-COVID positive(30)	TB positive(30)
1	Age	10.5(2-16)	6.5(2-17)
2	Male	12(30)	13(30)
3	Female	18(30)	17(30)
4	Weight (median)	25.5 (14.5-73.7)	21.6 (11.6-43.6)
5	Height	136 (104-170)	136 (96-157)
6	MUAC	17.5 (13.5-30)	18 (12.5-24)
7	TB Contact	9 (30%)	21(70%)
8	Previous TB History	9(30%)	2(6.6%)
Current TB status			
9	TB(Confirmed)/ TB(Un confirmed)	10(33.3%)	12(40%)
10	Type	8 PULMONARY 2 EXTRA PULMONARY	12 PULMONARY
Symptoms			
11	Fever	12(40%)	14(46.6%)
12	Cough	16(53.3%)	20(66.6%)
13	Shortness of breath	4(13.3%)	8(26.6%)
14	Loss of weight	13(43.3%)	18(60%)
15	Lack of energy	7(23.3%)	13(43.3%)
16	Vomiting	5(16.6%)	11(36.6%)
17	Abdominal pain	6(20%)	7(23.3%)
18	Headache	2(6.66%)	4(13.3%)
19	Loss of appetite	7(23.3%)	11(36.6%)
20	Night sweats	1(3.33%0	2(6.66%)
Investigations			
21	TST	23(76.6%)	24(80%)
22	HIV Status	0	1(3.33%)
23	CXR	12(40%)	17(56.6%)
24	CBNAAT	4(13.3%)	0
COVID IgG spike			
26	IgG(S) BL	91.32 (14.7-383.75) 30POSITIVE (100%)	0.65 (0.14-9.69)
27	IgG(S) M3	125.7 (12.59-481.52) 30POSITIVE (100%)	1.7 (0.22-77.36) 4POSITIVE (13.3%)
28	IgG(S) M6	33.84 (2.9-550.05) 20POSITIVE (66.6%)	0.53 (0.25-57.35) 6POSITIVE (20%)

### SARS-CoV-2 testing

Serological tests for antibodies against the viral Spike protein IgG (S) and membrane protein IgM were performed using the YHLO iFlash 1800 Chemiluminescence Immunoassay Analyzer using the iFlash-SARS-CoV-2 IgG (S). According to the manufacturer’s specifications, concentrations of IgG and IgM equal to or exceeding 10.00 AU/mL were deemed positive.

### Measurement of complement proteins and complement regulatory proteins

Systemic levels of complement proteins such as C1q, C2, C3, C3b/iC3b, C4, C4b, C5, C5a, and mannose-binding lectin (MBL), along with complement regulatory proteins like factor B, factor D, factor H, and factor I, were assessed using bead-based multiplex complement assay kits from Luminex Corporation. The lowest detection limits for each protein were as follows: C2 at 1.4 ng/mL; C4b at 1.4 ng/mL; C5 at 2.7 ng/mL; C5a at 4.1 ng/mL; complement factor D (adipson) at 0.07 ng/mL; MBL at 0.1 ng/mL; complement factor I at 0.7 ng/mL; C1q at 0.1 ng/mL; C3 at 0.3 ng/mL; C3b/iC3b at 8.2 ng/mL; C4 at 0.6 ng/mL; complement factor B at 0.1 ng/mL; and complement factor H at 0.04 ng/mL.

### Statistical analysis

Statistical analyses were performed using GraphPad Prism version 10 (GraphPad Software, CA, USA). Geometric means (GM) were used to summarize central tendencies. Differences between the CoV2^+^ and CoV2^-^ groups were evaluated by non-parametric *Mann-Whitney U test*, with Holm’s correction for multiple comparisons. The test was chosen due to the non-normal distribution of complement protein levels and the modest sample size and a *P* < 0.05 was considered statistically significant. Complement data across different time points were further examined using Canonical discriminant analysis (CDA). This was employed as an exploratory multivariate approach to capture coordinated, pathway-level differences in complement activation across predefined time points and SARS-CoV-2 serostatus groups. CDA is well suited for identifying composite immune signatures and temporal separation patterns rather than testing individual longitudinal effects was performed using JMP 17. Spearman correlation analysis was used to assess monotonic associations between SARS-CoV-2 IgG antibody levels and complement proteins at each time point independently, given the non-parametric nature of the data and the study’s focus on immune–immune interactions rather than predictive modelling. Heatmap visualizations were generated using R statistical software version 4.4.2. R-based analyses were conducted through RStudio version 2026.04.0 + 526.

## Results

### Demographics of study population

This study included 60 children, with a median age of 8.5 years. Of these, 23 (38%) were male. Among the 60 children, 22 children (36%) had microbiologically confirmed TB, which included 20 PTB and 2 EPTB. All were HIV negative, BCG-vaccinated at birth and tested negative for SARS-CoV-2 RT-PCR. (11) (**Table 1**).

### Altered complement protein levels in TB children with SARS-CoV-2 seropositivity

**Figure 1:**
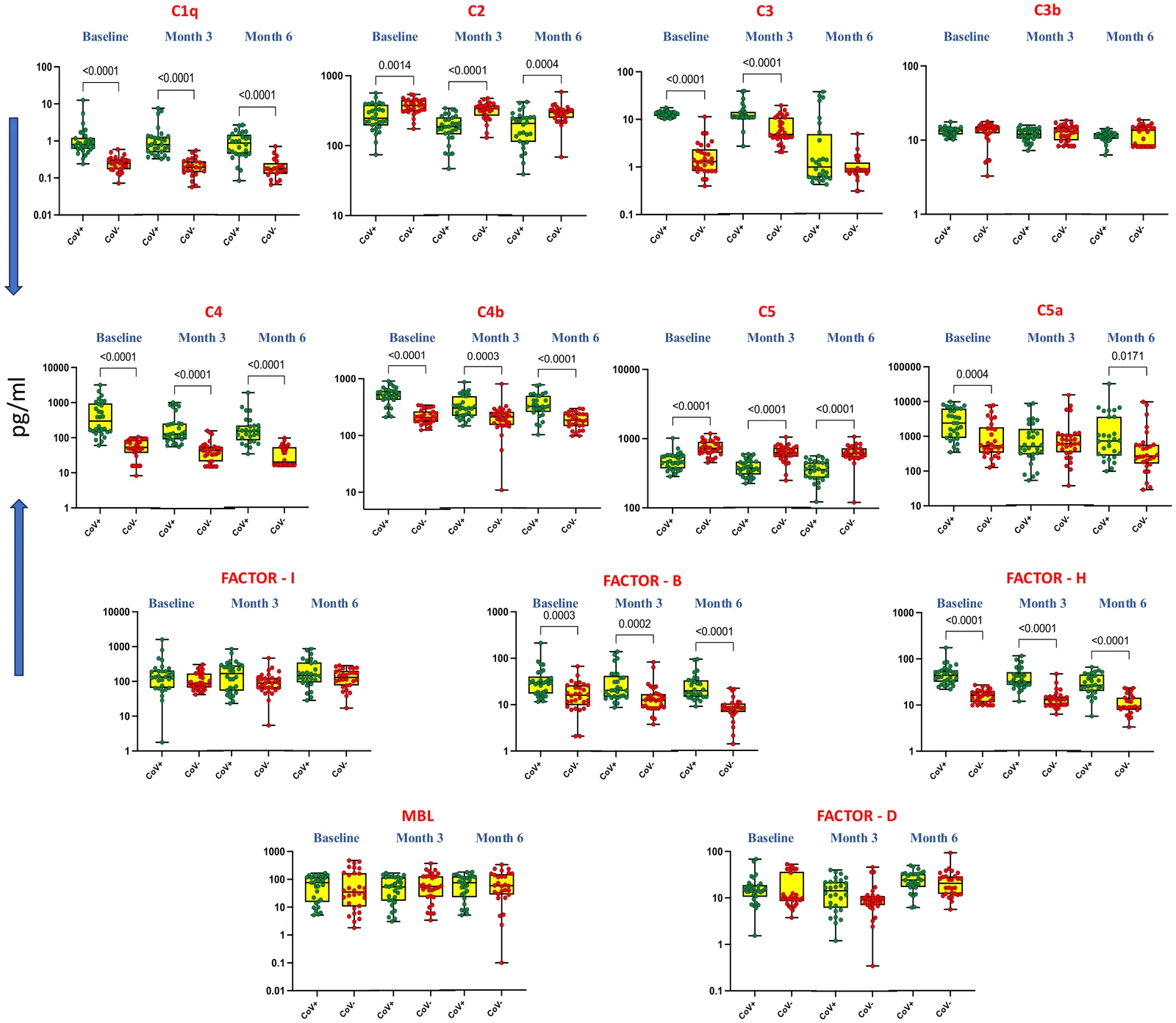
CoV2+ results in elevated plasma levels of complement proteins. The plasma levels of complement proteins were measured in CoV2+ (n = 30) and CoV2- (n = 30) children with TB disease at **(A)** Baseline, **(B)** Month 3, and **(C)** Month 6. Data are presented as scatter plots, with each circle representing a single individual. P values were calculated using the non-parametric Mann Whitney U test, with Holms correction for multiple comparisons.

To determine differences in the complement protein responses of TB children with or without serological evidence of prior SARS-CoV-2 exposure, we measured the plasma proteins of C1q, C2, C3, C4, C4b, C5 and C5a during TB treatment. As evidenced in **Figure 1**, TB diseased children with SARS-CoV-2 seropositivity (CoV2^+^) had higher levels of C1q, C3, C4, C4b and C5a at baseline than TB diseased children without (CoV2^-^). Levels of C1q, C3, C4, C4b, and C5a continued to be high in CoV2^+^ group at month 3, while At month 6, differences between groups were attenuated for several complement proteins. However, circulating C2 and C5 levels remained significantly lower in CoV2^+^ children than in CoV2^-^ children at baseline, month 3, and month 6. Expression of regulatory and recognition components of the complement cascade were also significantly different between groups TB diseased children with SARS-CoV-2 seropositivity (CoV2^+^) and TB diseased children without seropositivity (CoV2^-^). Higher levels of Factor B and Factor H were evident in CoV2^+^ children compared to CoV2^-^ children at baseline. Similar pattern was noted in month 3 and month 6 particularly for Factor B and Factor H. These results supports the premise that children with TB and SARS-CoV-2 exposure (CoV2^+^) exhibit increased activation of the classical and alternative complement activation pathway which likely results in an increased inflammatory influence. The consolidated p-values are presented in **Supplementary Table 1**.

### Longitudinal complement profiling via canonical discriminant analysis

**Figure 2:**
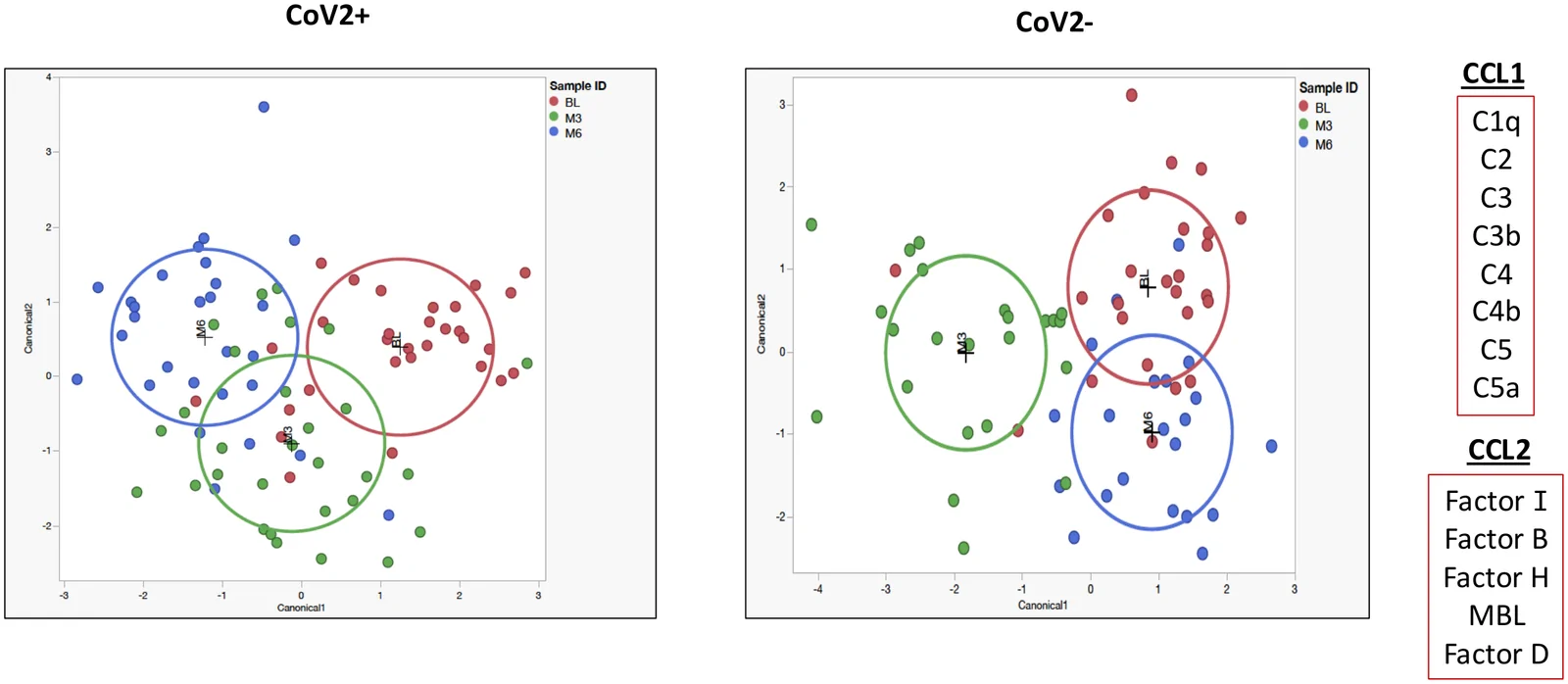
Complement proteins are engaged in TB children with and without CoV2 infection during baseline and post-treatment. Canonical discriminant analysis (CDA) was performed to evaluate whether the cytokine CCL1 could discriminate between samples collected at baseline (M0), Month 3 (M3), and Month 6 (M6). The analysis was conducted using the defined grouping factor based on study time points. Data are presented as dot plots, with each dot representing an individual participant.

We used canonical discriminant analysis to explore temporal patterns of complement activity in children with TB according to SARS-CoV-2 serostatus. The complement proteins were grouped into two functional communities, CCL1 (classical pathway proteins: C1q, C2, C3, C3b, C4, C4b, C5, C5a) and CCL2 (alternative and surface lectin pathway proteins and regulators: Factor I, Factor B, Factor H, MBL, Factor D). The results (**Figure 2**) indicate that CoV2^-^ children temporally separate by time point (baseline (BL), month 3 (M3), and month 6 (M6)), which shows that the complement activity of CoV2^-^ children was mobilized and coordinated over time in a progressive manner, not influenced by an activation via virus. The CoV2^+^ group demonstrated a response across time points that overlapped, suggesting that virus induced a protracted and dysregulated recovery of complement activation. Overall, the results illustrate the power of CDA as exploratory visualization of multivariate complement patterns, and the potential of complement profiling as a biomarker of immune recovery after SARS-CoV-2 infection.

### Correlation analysis of complement protein levels over time in individuals with and without SARS-CoV-2 seropositivity

**Figure 3:**
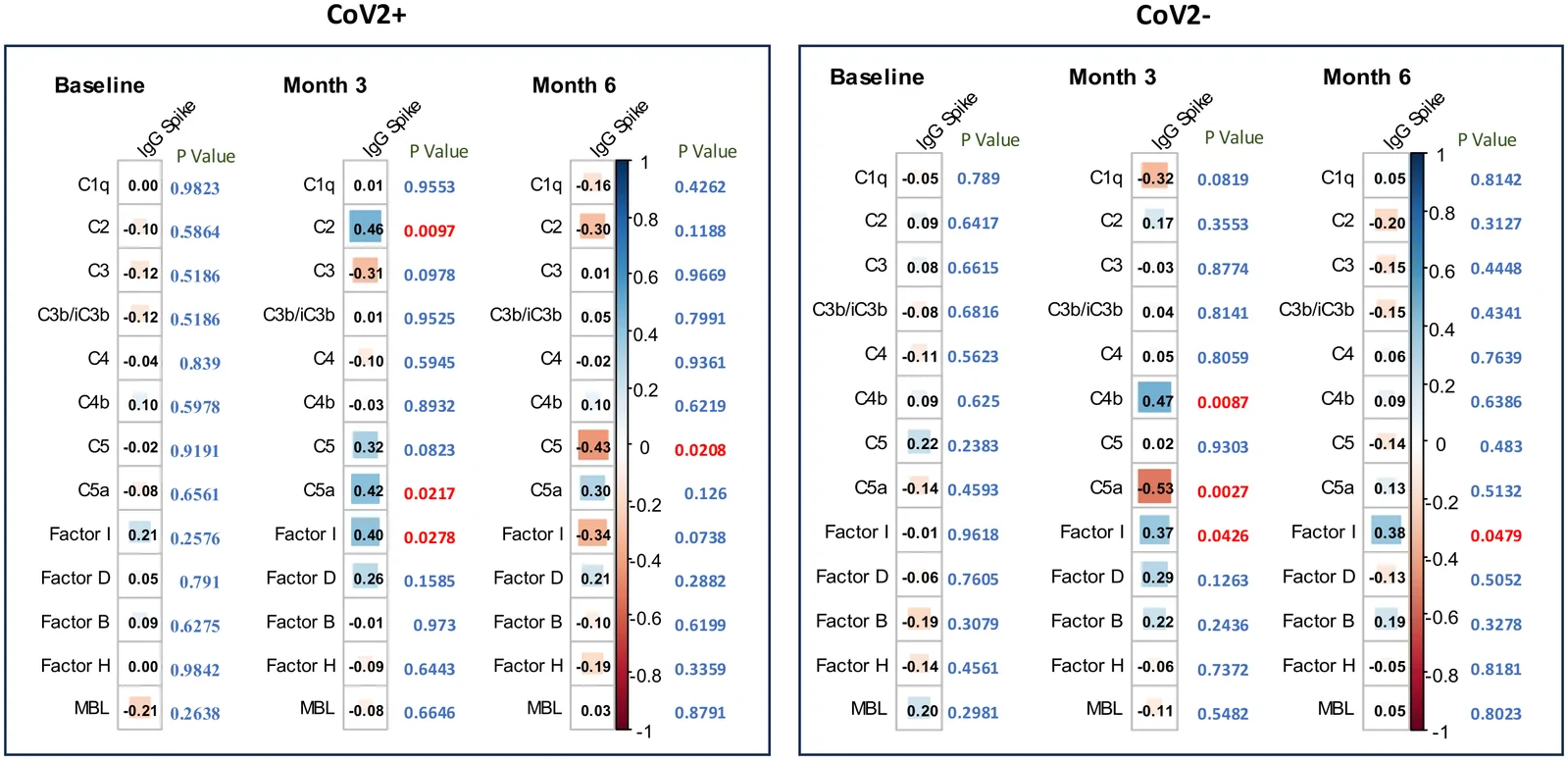
Relationship between complement proteins and IgG spike protein levels. A multiparametric matrix correlation plot of complement proteins and IgG spike protein levels in all individuals of CoV2+ and CoV2- at baseline (M0), Month 3 (M3), and Month 6 (M6). Spearman’s correlation coefficients are visualized by color intensity. P values and Spearman r values are ordered by hierarchical clustering. In the correlation plot, the box size represents the correlation value ranging from -1 to +1; larger sizes indicate stronger correlations. Blue color represents positive correlation, while red color represents negative correlation. Statistically significant p-values are denoted as follows: *p<0.05, **p<0.01, ***p<0.001.

We aimed to investigate the longitudinal relationship between complement system activation and SARS-CoV-2-specific humoral responses by analyzing correlations between IgG Spike antibody levels and individual complement proteins at baseline, month 3 (M3), and month 6 (M6) in both CoV2^+^ and CoV2^−^ individuals. Spearman’s correlation coefficients were calculated, and results were visualized using heat map color gradients to represent the strength and direction of association (**Figure 3**). In the CoV2^+^ group, a significant positive correlation was observed between IgG Spike and C2 (r = 0.46, p = 0.0097), C5a (r = 0.42, p = 0.0217) and Factor I (r = 0.40, p = 0.0278) at M3, as well as C5 (r = –0.43, p = 0.0208) at M6. Conversely, the CoV2− group exhibited distinct negative associations at M3 between IgG Spike and C5a (r = –0.53, p = 0.0027), and positive correlations with C4b (r = 0.47, p = 0.0087) and Factor I (r = 0.37, p = 0.0426). At M6, a significant inverse correlation was detected between IgG Spike and Factor I (r = 0.38, p = 0.0479). The results above demonstrate that the relationship between antibody titres and complement activation varies based on prior infection history with SARS-CoV-2. CoV2^+^ exhibited both direct and inverse associations with specific complement proteins, while CoV2^-^ exhibited a stricter pattern of regulation, suggesting altered or re-set homeostasis following SARS-CoV-2 exposure. The consolidated p-values are presented in **Supplementary Table 2**.

## Discussion

The study provides strong evidence for important alterations in the complement system in TB Children with serological evidence of prior SARS-CoV-2 exposure. Our data show that children with serologic evidence of prior exposure to SARS-COV-2 (CoV2^+^) demonstrate a persistent and exaggerated response of the complement response compared to children without SARS-CoV-2 exposure (CoV2^-^) throughout the TB treatment period. These findings indicate substantial alterations in the immune landscape, highlighting a potential role of SARS-CoV-2 in modulating innate immune pathways. The observed complement alterations may reflect immunological changes associated with TB in the context of prior SARS-CoV-2 exposure, without implying direct effects on clinical outcomes. (12). The higher baseline levels of complement classical pathway proteins (C1q, C2, C3, C4, C5, and C5a) in CoV2^+^ children suggests that SARS-CoV-2 may sensitize, or dysregulate, to complement system activation, even in the absence of detectable acute SARS-CoV-2 infection by RT-PCR. These higher levels are consistent with previous studies that have implicated which results in immune activation for convalescent COVID-19 cases, especially among children, with subclinical or even asymptomatic infections (5, 12). We previously demonstrated that activation of the complement system is linked to the pathogenesis of MIS-C and COVID-19 in children (10). In addition it is also been reported that persistent complement activation observed in CoV2^+^ children with TB parallels reports from pediatric MIS-C and post-acute COVID-19 studies, where prolonged activation of innate immune pathways including complement and cytokine signaling has been implicated in immune dysregulation even after resolution of acute infection (13) The elevated complement levels observed in this study further support our earlier findings, indicating that prior SARS-CoV-2 exposure may trigger a sustained and amplified complement activation.

The elevated levels at month 3 and partial resolution at month 6 may be an indication of prolonged recovery, or low-grade inflammation that persisted in the coinfected subjects. Importantly, regulatory proteins, Factor B and Factor H, which were higher levels during follow up in CoV2^+^ children could be markers of compensatory reactions to allow regulation of the ongoing complement activation. However, this compensatory reaction may have been inadequate or delayed which could explain triggering inflammation, and impaired resolution of TB disease (14).

Complement proteins, including C5a, function as potent anaphylatoxins that recruit immune cells and promote cytokine release. The persistent elevation of C5a in CoV2^+^ children is concerning, as it may lead to tissue damage or worsen lung pathology related to tuberculosis (15). In cases of coinfection, the shared tropism targeting pulmonary tissues and the immune pathology associated with TB and SARS-CoV-2 may be even more challenging if it induces a new inflammatory space that suppresses granuloma formation or sustains mycobacterial infections. Complement-mediated opsonisation plays a significant role in the cleaning of Mtb; however, it may be attenuated in CoV2^+^ children, potentially leading to inefficient pathogen clearance or immune re-direction. The application of canonical discriminant analysis (CDA) offered a new systems-level understanding, showing that TB children with no past SARS-CoV-2 exposure are more temporally and functionally ordered in their immunological response. The temporal overlap of complement-associated inflammatory profile, in CoV2^+^ children suggests poorly regulated or asynchronously resolving complement responses. Consistent with our findings of dysregulated immunologic recovery in CoV2^+^ children, similar patterns have been reported more broadly in the adult COVID-19 literature, where delayed normalization of complement response has been associated with worse clinical outcomes. (16). Above all, CDA offers recognition of complement profiling as a potential immune recovery and disease monitoring biomarker, especially when traditional diagnostics are ineffective.

Our correlation analysis further underscores the functional pertinence of complement SARS-CoV-2 seropositivity may be associated with altered complement activation patterns. The discordant relationships observed between spike IgG antibody levels and complement proteins in CoV2^+^ in comparison to CoV2^-^ cohorts suggest that humoral and innate responses following infection operate very differently. Intriguingly, the positive correlation between spike IgG and C2, C5a, and Factor I at Month 3 in CoV2^+^ children implies that antibody-mediated complement activation may persist well into TB therapy. The inverse correlations described in CoV2^-^ children seem to reflect a more expected homeostatic resolution to seem T-cell mediated if at all. These patterns suggest that SARS-CoV-2 infection is not only coincident but may also alter immune trajectories in children with TB (17). These findings have meaningful implications both for the public health community and for clinicians. First, it may be possible that children with prior exposure to SARS-CoV-2 constitute a vulnerable sub-population of children with TB who may require heightened surveillance or modifiable treatment approaches. Second, specifically targeting the complement pathway therapeutically–possibly with selective inhibition of C5a or modulating factor H–may serve as a new adjunctive therapy to limit excessive inflammation and improve outcomes in affected children (15). However, this must be weighed carefully to not impede the necessary complement response to Mtb.

This work also reflects the wider challenges in coinfection research. Our findings provide mechanistic evidence, albeit limited by sample size and lack of direct clinical outcome data linking complement profiles to natural progression of TB disease severity or recovery. Future work should more definitively examine the causal role of complement dysregulation in the complement response during TB-COVID coinfection with larger, longitudinal cohorts, and further investigate the local activity of complement. Integrating transcriptomic or proteomic analyses would also foster an understanding of the upstream regulators and downstream effects of dysregulated complement signalling. This study has several limitations that warrant consideration. Potential confounding factors, including nutritional status, age between the study groups effects of BCG-induced immune imprinting, and exposure to other childhood infections, were not systematically assessed and therefore could not be included as covariates in the analyses. These factors may influence complement activity and complement response in children with tuberculosis. Future studies incorporating standardized nutritional assessments, detailed evaluation of concurrent or prior endemic coinfections, and stratification based on vaccine-induced complement response will be essential to more clearly define the independent association between SARS-CoV-2 seropositivity and complement activation in pediatric TB.

In summary, this study demonstrates that TB Children with serological evidence of prior SARS-CoV-2 exposure results in an exaggerated complement activation suggesting a dysregulated complement response that could complicate management of TB disease. Our results suggest the value of immune profiling in co-infected children and support the complement system as both a biomarker and therapeutic target in managing TB–COVID-19 interactions. Insights such as these are imperative in designing targeted interventions for the most vulnerable children suffering from two concurrent infectious disease burdens, particularly in low- and middle-income countries where future coinfection rates will continue to be significant.

## Data Availability

The original contributions presented in the study are included in the article/**Supplementary Material**. Further inquiries can be directed to the corresponding author.
