# Supplementary material for: Profiling complement proteins in pediatric tuberculosis children with or without serological evidence of prior SARS-CoV-2 exposure
Source: Front Immunol. 2026 Aug 7;17:1761877. doi: 10.3389/fimmu.2026.1761877 (PMC13493294; doi:10.3389/fimmu.2026.1761877)
Supplement: Supplementary file 1 [file DataSheet1.pdf]

Supplementary Table 1 : p Values shown in the Figure 1

Analyte	P value		
	BL<br>CoV+ vs CoV-	M3<br>CoV+ vs CoV-	M6<br>CoV+ vs CoV-
**C1q**	<0.0001	<0.0001	<0.0001
**C2**	0.0014	<0.0001	0.0004
**C3**	<0.0001	<0.0001	0.6557
**C3b**	0.0804	0.2859	0.2119
**C4**	<0.0001	<0.0001	<0.0001
**C4b**	<0.0001	0.0003	<0.0001
**C5**	<0.0001	<0.0001	<0.0001
**C5a**	0.0004	0.6865	0.0171
**Factor I**	0.1091	0.0906	0.3242
**Factor D**	0.1229	0.0906	0.2856
**Factor B**	0.0003	0.0002	<0.0001
**Factor H**	<0.0001	<0.0001	<0.0001
**MBL**	0.7635	0.4492	0.8263

Supplementary Table 2 : Correlation analysis p Values shown in the Figure 3

CoV+

Analyte	P value		
	BL	M3	M6
**C1q**	0.9823	0.9553	0.4262
**C2**	0.5864	0.0097	0.1188
**C3**	0.5186	0.0978	0.9669
**C3b**	0.5186	0.9525	0.7991
**C4**	0.839	0.5945	0.9361
**C4b**	0.5978	0.8932	0.6219
**C5**	0.9191	0.0823	0.0208
**C5a**	0.6561	0.0217	0.126
**Factor I**	0.2576	0.0278	0.0738
**Factor D**	0.791	0.1585	0.2882
**Factor B**	0.6275	0.973	0.6199
**Factor H**	0.9842	0.6443	0.3359
**MBL**	0.2638	0.6646	0.8791

CoV-

Analyte	P value		
	BL	M3	M6
**C1q**	0.789	0.0816	0.8142
**C2**	0.6417	0.3553	0.3127
**C3**	0.6615	0.8774	0.4448
**C3b**	0.6816	0.8141	0.4341
**C4**	0.5623	0.8059	0.7639
**C4b**	0.625	0.0087	0.6386
**C5**	0.2383	0.9303	0.483

**C5a**	0.4593	0.0027	0.5132
**Factor I**	0.9618	0.0426	0.0479
**Factor D**	0.7605	0.1263	0.5052
**Factor B**	0.3079	0.2436	0.3278
**Factor H**	0.4561	0.7372	0.8181
**MBL**	0.2981	0.5482	0.8023
